# Lupus-Induced Accelerated Heart Failure in a Young African American Female: Cardiovascular and Systemic Complications of Noncompliance to Maintenance Therapy and the Social Determinants of Cardiovascular Disease

**DOI:** 10.7759/cureus.51819

**Published:** 2024-01-07

**Authors:** Ikpechukwu J Okorie, Edmund Appiah-Kubi, Philipa Owusu-Antwi, Evans Takyi, Derek Ugwendum, Annmarie Fernando, Muhammed Atere, Jay Nfonoyim

**Affiliations:** 1 Internal Medicine, Richmond University Medical Center, New York, USA; 2 Internal Medicine, Allegheny Health Network, Pittsburgh, USA; 3 Psychiatry, Richmond University Medical Center, New York, USA; 4 Internal Medicine, American University of Antigua, New York, USA; 5 Medicine, Richmond University Medical Center, New York, USA; 6 Pulmonary and Critical Care, Richmond University Medical Center, New York, USA

**Keywords:** hydroxychloroquine, left ventricular hypertrophy (lvh), heart failure, social determinants of health (sdoh), cardiomyopathy, systemic lupus erythema

## Abstract

Systemic lupus erythematosus (SLE) is an autoimmune inflammatory disorder characterized by dysregulations of the immune system with intermittent and remitting symptoms. SLE affects multiple organs and systems, including the cardiovascular system. This condition is associated with an increased risk of cardiovascular disease, particularly in younger patients. Our case report describes a patient who rapidly developed structural, functional, and electrophysiological cardiac abnormalities due to lupus-induced cardiomyopathy. The accelerating cardiac events were the result of medication noncompliance. Myocarditis and other potentially fatal cardiac complications associated with SLE have been the subject of numerous studies. This presentation appears to be the first to emphasize the rarity of lupus-induced cardiomyopathy, the importance of treatment adherence, the adverse cardiac effects of targeted therapeutic interventions, and the influence of social determinants of cardiovascular health on a patient's prognosis.

## Introduction

Systemic lupus erythematosus (SLE), a chronic autoimmune illness causes widespread inflammation and tissue damage in the affected organs due to an inappropriate immune response [1]. Despite the disease's clinical heterogeneity and different pathogenetic processes, SLE patients' illness is not fully explained by the multiple diverse pathways of autoimmune mechanisms [1]. SLE patients have a three to fourfold higher risk of cardiovascular events and mortality than others with similar risk factors, such as major adverse cardiovascular events and subclinical atherosclerosis [2].

About half of SLE patients suffer cardiac problems and have varied cardiac presentations. The most common cardiac consequence is myocarditis, which can impair systolic or diastolic function [3]. Some cardiac complications reported are pulmonary hypertension, valvular heart disease, pericarditis, pancarditis, and premature atherosclerosis [3,4]. However, multiple concurrent cardiac dysfunctions (including QT prolongation, moderate to severe septal thickness, moderate posterior left ventricular thickness and mild hypokinesis, moderate right ventricular (RV) dilatation/RV volume overload, moderately dilated left atrium, severe right atrial dilatation, moderate thickening and calcification of anterior mitral leaflet, moderate mitral regurgitation, and moderate mitral annular calcification) and associated systolic and moderate diastolic dysfunction are uncommon. These cardiac findings significantly increase morbidity and mortality in SLE patients.

Hormonal, immunological dysregulation, and genetic vulnerability are suspected as attributing causes [3]. Numerous case reports dominate the literature for SLE describing either atypical presentation of SLE or limited cardiac complications in single patients [2,3]. The present case describes numerous complications of uncontrolled SLE flares, the implication of medication noncompliance and social determinants of cardiovascular disease in SLE. Myocardial inflammation causes fibrosis, scarring, and cardiac dysfunction in lupus-induced cardiomyopathy. SLE disproportionately affects women of reproductive age, with an incidence ratio of one in 10 compared to individuals with the same levels of traditional risk factors [4]. Most people with SLE cardiomyopathy do not have symptoms; however, they are at high risk of developing heart failure and arrhythmias silently [4]. SLE cardiomyopathy is best diagnosed by endomyocardial biopsy [4]. Cardiac magnetic resonance is an alternative modality, primarily due to its excellent spatial and temporal resolution and fast capture [5].

The rarity of SLE cardiomyopathy complicates treatment. Several studies have associated hydroxychloroquine (HCQ) with negative adverse effects on the heart. On the other hand, the use of this immunoregulatory therapy has been shown to reduce SLE flare-ups and improve patient mortality [6]. This case report shows rapid cardiac problems and numerous organ failures in a young African American female with a disease flare-up after years of poor compliance with treatment. The case also explores medication adherence, the cardiac risks of specialized treatment, social determinants of cardiovascular health, and the correlation between depression, anxiety, and SLE.

## Case presentation

A 24-year-old African American female presented to the emergency department (ED) with diffuse body aches for two days, fatigue, dizziness, dyspnea, and failure to perform routine daily activities. She had a history of SLE (non-compliant with her medication), lupus nephritis, asthma, and seizures. She also had a social history of occasional marijuana and alcohol use but denied recent use of recreational drugs. On the morning of the presentation, she felt a spinning sensation, vomited about four times, and had limited oral tolerance. She mentioned temporary loss of consciousness suspected to be a seizure episode.

**Figure 1:**
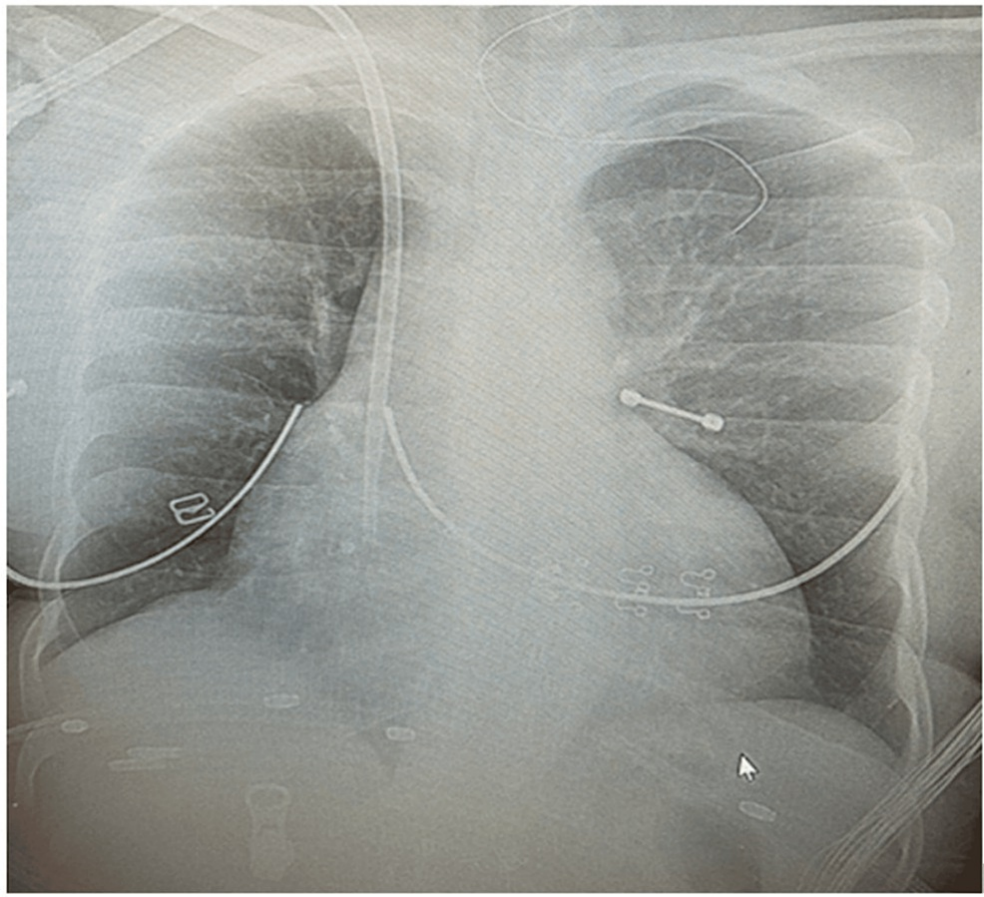
Chest X-ray in the current presentation (2023) showing cardiomegaly with dilation of the atrium. There is non-specific ground opacification with infectious or inflammatory etiology in the lungs.

Upon arrival in the ED, she was found to be hypoxic and hypotensive. Physical exam was significant for bibasilar lung crackles and mildly distended tensed abdomen. Heart sounds were regular, no murmur was heard, and jugular distention was seen. Further physical examination was negative for leg edema, fever, clubbing, lymphadenopathy, or cyanosis. During further questioning, she reported experiencing dyspnea on exertion but denied any cough or nocturnal dyspnea. She was placed on non-rebreather mask oxygen supplementation and was later transitioned to nasal cannula as her oxygen saturation gradually improve. Hypotension resolved with a one-liter bolus of normal saline and subsequent gentle hydration to maintain mean arterial pressure greater than 65. Other emergency management courses include morphine 2mg IV once for body pain, Keppra 1000 mg IV once for unwitnessed seizure at home. Chest x-ray showed cardiomegaly (Figure 1). CT abdomen and pelvis showed moderate to large ascites. ED laboratory work-up showed elevated pro-brain natriuretic peptide (pro-BNP), creatinine, and mildly elevated potassium (Table 1).

**Table 1:** Laboratory report during the course of hospital stay ANA=anti nuclear antibody; NTpro BNP=N-terminal pro-hormone of brain natriuretic peptide; GFR=glomerular filtration rate; MPO=myeloperoxidase; DsDNA ab=double stranded deoxyribonucleic acid antibody

Test	Value	Reference
C-Reative Protein	1.2	<1.2
Erythrocyte sedimentation rate	26	0 - 20
Hemoglobin A1C	5.4	<5.7
Brain natriuretic peptide	>5000	<100
NT-pro-B BNP	175000	0-125
Troponin 1 high-sensitivity	22.3	0 - 34
White blood cell	5.1	4.0-11.2	
Hemoglobin	12.3	11.2-15.7	
Platelet count	215	150-400	
Blood urea nitrogen	51	7-18	
Creatinine	6.23	0.55-1.02	
GFR	8	>60	
Sodium	136	136-145	
Potassium	5.2	3.5-5.1	
Phosphorus	5.6	2.4-5.1	
Magnesium	2.25	1.60-2.60	
Calcium	7.1	8.3-10.6	
ANA screen	Positive	Neg	
ANA titer	1.320	<1.40	
ANA pattern	AC-2,4,5,29	-	
Antiproteinase 3 FEIA c/o 1.9	<1.0	<1.0	
Anti-MPO Ab	<1.0	<1.0	
DsDNA ab	Positive	-	
Anti-DNA antibody titer	1.320>1.80	<1.40 negative	
Complement C3	130	83-193	
Complement C4	32	15-57	

**Figure 2:**
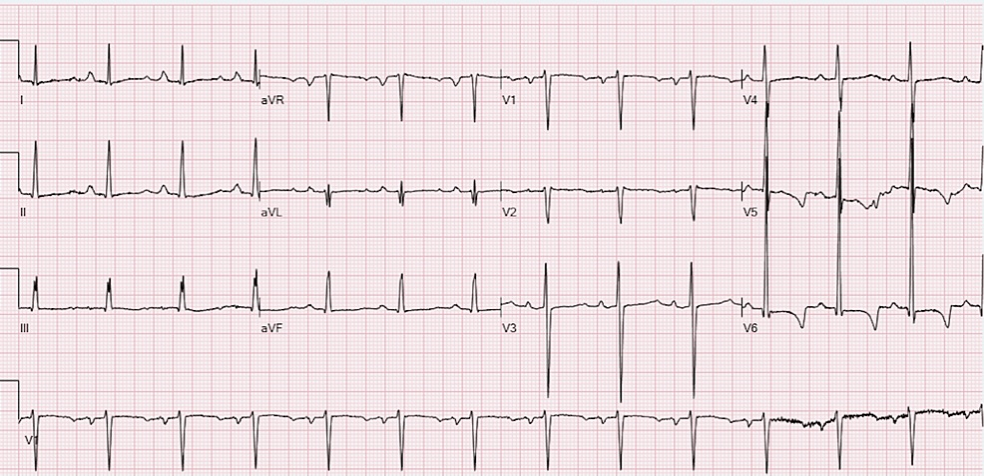
Normal sinus rhythm with possible left atrial enlargement, left ventricular hypertrophy, ST & T wave abnormality with prolonged QT of 573

**Figure 3:**
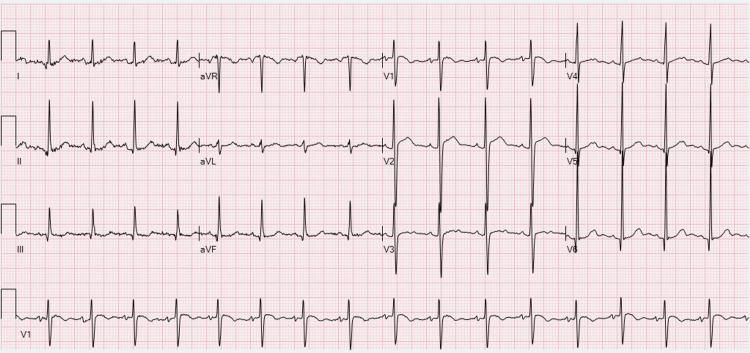
Normal EKG and QT two years prior to current presentation

Electrolytes were corrected and a nephrologist was consulted for possible hemodialysis and chronic kidney disease management. EKG shows normal sinus rhythm, high voltage QRS, nonspecific ST & T wave abnormality, and prolonged QT (Figure 2) compared to normal QTC in the EKG taken two years prior to this presentation (Figure 3). 

**Figure 4:**
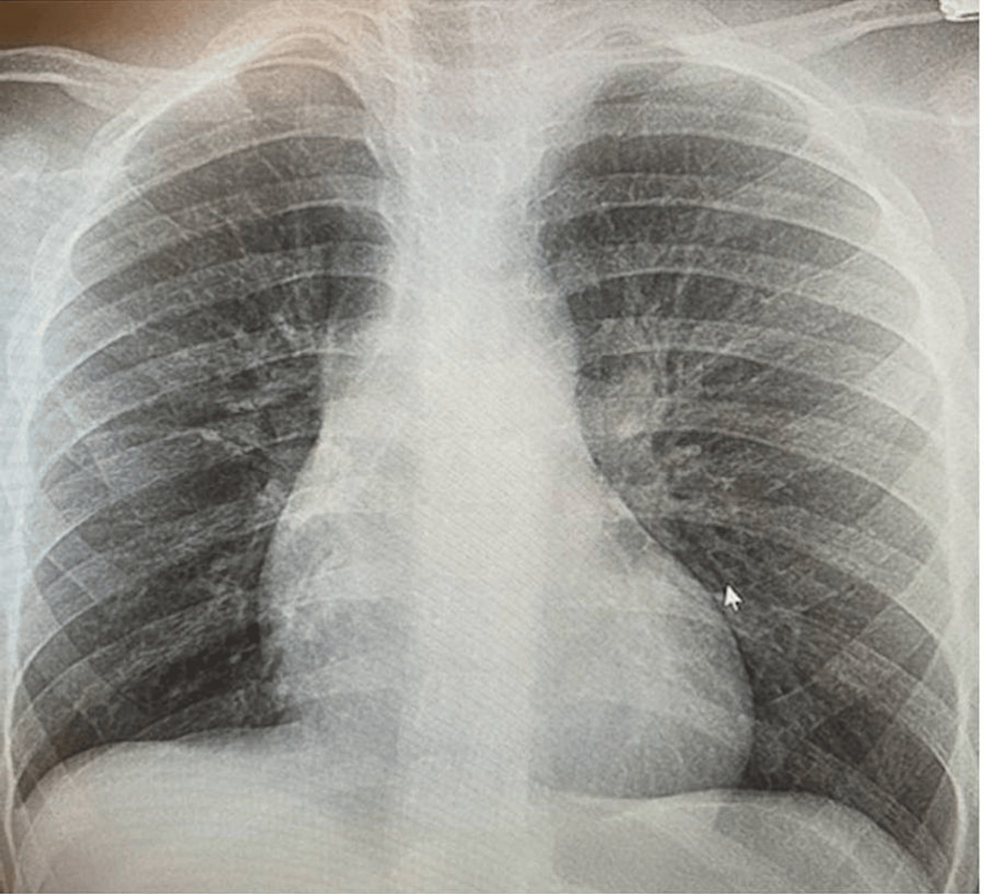
Chest X-ray from 2020 displaying no confluent infiltrate or effusion.

The patient was diagnosed with SLE during pregnancy at the age of 20. She denied any family history of autoimmune disease. Upon diagnosis, she was placed on lupus medication, hydroxychloroquine 200 every 12 hours by her rheumatologist. The patient stated that she discontinued the medication over two years, and the last refill according to medication reconciliation was in early 2021. It was also unsure if she had been compliant prior to the time of discontinuation. Upon evaluation, she was calm, polite, and cooperative but appeared depressed about her condition. She stated she knew that she had to follow up with her rheumatologist but had not been compliant due to lack of motivation ''as there is no absolute cure for my condition''. She was diagnosed with possible SLE flare and congestive heart failure. Other concerned findings include moderated ascites, pulmonary congestion, and elevated high sensitive BNP. Chest x-ray showed an enlarged cardiac silhouette (Figure 1), a sequela thought to be linked to the chronic nature of her disease, compared to changes seen in prior chest imaging three years ago (Figure 4).

**Figure 5:**
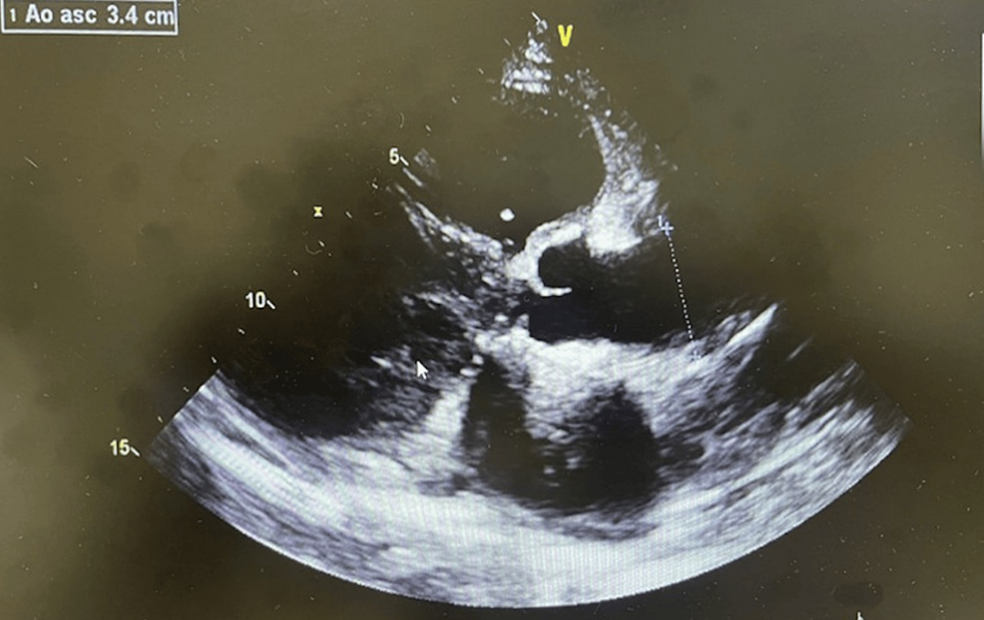
Echocardiogram showing ventricular dilation with ventricular wall hypertrophy.

Due to elevated BNP, low blood pressure, ascites observed, and worsening cardiomegaly with pulmonary edema seen in chest X-ray, echocardiography was ordered. Echocardiographic findings were indicative of several worsening cardiac disease processes (Figure 5, Video 1).

**Video 1:** Echocardiogram video showing ventricular systolic dysfunction, left ventricular hypertrophy, and severe tricuspid regurgitation

The echocardiography report was compared to a prior study two years ago. The differences are shown in Table 2.

**Table 2:** Comparison of echocardiography reports LVEF= left ventricular ejection fraction; SLE=systemic lupus erythematosus

Findings	2023	2021
Indication	History of low LVEF	Dyspnea, Heart failure, Hypertensive urgency, End stage renal disease, SLE
Study Quality	The echo study is of adequate quality	Adequate quality
Rhythm	Normal sinus rhythm	Normal sinus rhythm
Left Ventricle	- Left ventricular size: Normal - LVEF: 35% - Moderate left ventricular hypertrophy - Ventricular septal thickness: Moderately to severely increased - Paradoxical movement of ventricular septum (c/w right ventricle volume overload) - Left ventricle posterior wall thickness: Moderately increased - Left ventricle posterior wall: Mildly hypokinetic	- Normal size - Mild LV global hypokinesis - LVEF approximately 45-50% - Moderate left ventricular hypertrophy - Moderately increased ventricular septal thickness - Mild to moderately hypokinetic ventricular septum - Moderately increased LV posterior wall thickness - Mild to moderately hypokinetic left ventricle posterior wall
Right Ventricle	- Moderately enlarged - Normal right ventricular systolic function	Normal size and systolic function
Left Atrium	Moderate left atrial dilatation	Moderately dilated
Right Atrium	Severe right atrial dilatation	Normal size
Aortic Root	Aortic valve appears normal	Normal
IVC (Inferior Vena Cava)	Right ventricle volume overload	Normal with estimated right atrium pressure of 3 mmHg
Mitral Valve	Moderate mitral regurgitation - Moderate mitral annular calcification	- Increased E point to septal separation - Mild-to-moderate mitral regurgitation - Mild mitral annular calcification
Tricuspid Valve	Severe tricuspid regurgitation	Moderate tricuspid regurgitation
Pulmonic Valve	Mild pulmonic regurgitation	Trace (physiologic) pulmonic regurgitation
Pericardium	Trivial pericardial effusion vs. subepicardial fat	Trivial posterior echo-free space consistent with small pericardial effusion or subepicardial fat

The patient was dialyzed and managed for chronic renal impairment by the nephrology team. With the new and worsening cardiac findings, cardiology was consulted, and the patient started on aspirin, high-dose statin, metoprolol, and Lasix 40 mg IV. Nifedipine and clonidine for blood pressure control were continued following the return of blood pressure to baseline. The patient was started on steroids for possible lupus flare and a rheumatologist was consulted for further management and restarting of disease-modifying medications.

CT chest was done on the second day of the presentation and indicated chest infiltrate suspected to be pneumonia. Since the patient had episodes of syncope, she was under high suspicion for aspirated pneumonia, and antibiotics were administered for five days in this current admission.

The echocardiogram report showed several abnormal cardiac findings including heart failure with reduced ejection fraction (systolic dysfunction), diastolic dysfunction, cardiogenic pulmonary congestion, QTc prolongation, and valvular heart diseases. Due to these cardiac findings and hemodynamic instability, the patient was transferred to the telemonitoring floor for treatment. The cardiology team was involved in the care and recommended continuation of diuretics, the addition of angiotensin receptor-neprilysin inhibitor, beta blocker, and sodium-glucose transporter 2 inhibitors as tolerated. It was a Friday and as her case did not require urgent cardiac intervention, they recommended medical management and further evaluation for possible work-up the following Monday.

Since rheumatology was also consulted, she was advised to await in-patient rheumatological evaluation and outpatient follow-up afterwards. She was further managed by a multidisciplinary team on the floor. The following day, as care continued, the patient decided she wanted to leave against medical advice as her symptoms improved, to go take care of her dependent baby at home. An appointment was made to follow up with primary care (to be referred to psychiatry), cardiology, rheumatology, and nephrology outpatient. HCQ and other discharging medications were sent electronically to her pharmacy. She reiterated her understanding of the importance of compliance with her medications and follow-up with every team involved in her care and left. The patient was followed up two weeks later and she reassured compliance with her medications and out-patient follow-up with specialists involved in her care.

## Discussion

SLE is a chronic inflammatory disease that disproportionately occurs commonly in younger females and is highest in individuals of African American descent [2,7]. Although SLE can affect any organ, cardiovascular disease (CVD) seems to be a major cause of mortality for lupus patients [7,8]. Several studies have linked SLE to accelerated CVD in patients aged 15-45, compared to healthy individuals of similar age and gender [7]. Among other rheumatological diseases, SLE is one of the common autoimmune diseases associated with accelerated CVD as first reported by Urowitz et al. in 1976 [8]. Since then, more recent reports have demonstrated that the risk of CVD on average is two to tenfold higher in SLE patients when compared to the general population [9]. 

SLE can involve many different components of the heart including the pericardium, myocardium, valvular, coronary, and electrical system. Most reports describe patients with one or little lupus-induced cardiac disease or atypical clinical presentation, but the current report describes multiple cardiac effects of lupus affecting structural, functional, and electrical cardiac activities in a young African American, at a very young age, posing poor disease prognosis. These premature multiple cardiac diseases are thought to be due to increased SLE disease activity, associated complications, frequent flares, poor disease control, lupus-associated non-traditional CVD risk factors, and traditional CVD risk factors. There is also a link that few of the medications used for SLE management have some cardiac adverse effects that might worsen the cardiotoxic burden, although the probability of occurrence is rare [10,11]. Furthermore, increased disease activity leads to more complications and multi-organ disease.

In our case, long-term lack of disease control in our patient is indicated by frequent emergency visits for flares, non-compliance with lupus maintenance medications, uncontrolled blood pressure, and subsequent lupus nephritis. Lupus nephritis with poorly controlled hypertension, steroid use, and associated hyperglycemia during flares contributed to subsequent renal failure.CVD is a major cause of morbidity and mortality in chronic systemic inflammatory disease patients, and even worse outcomes in patients with chronic kidney disease, renal failure, and dialysis dependence. Increased lupus activity in our patient is also shown with elevated antinuclear antibody, lupus lung disease, suspected lupus cerebritis, seizure, and depressive mood. The cardiac dysfunction diagnosed with echocardiography was first discovered in 2021 during a lupus flare (Figure 5, Videos 1, Table 2). When compared to echocardiography obtained in 2023 following her ED visit, several worsening declines in cardiac functions were observed. The systolic function had deteriorated from the left ventricular ejection fraction range of 50-35%, along with other structural and valvular defects. As noticed in the initial blood work, there were laboratory signs of increased lupus activities as seen with positive ANA, anti-DsDNA antibody, and inflammatory markers. She initially followed up with her rheumatologist and was placed on HCQ 200 mg twice daily, but was not compliant for a long time.

Several reports have shown a significant slowing of disease activity and multiple organ protective benefits of disease-modifying agents [10,12-14]. The majority of SLE patients are not compliant with taking HCQ regularly. This is further demonstrated when serum drug cumulative levels in some SLE patients taking HCQ were sub-therapeutic. This indicates that many SLE patients are not compliant [15]. It is also unsure how long our patient was compliant with medication. Lupus leads to severe complications if not controlled by first reducing traditional risk factors and non-traditional risk factors. 

Treatment is necessary to mitigate disease and progressive organ dysfunction and complications. It will be important to understand the pathophysiology of disease processes to better understand the mechanism of choice of drugs used in the control of the disease process. The pathophysiology of lupus is not quite fully understood; however, reports have linked lupus to several mechanisms involving chronic inflammatory damage and fibrosis [5]. The pathophysiology of premature CVD in SLE is multifactorial and results from the attribution of traditional cardiac risk factors and the pro-inflammatory effect of SLE. Non-traditional risk factor involves lupus chronic inflammatory process involving multiple inflammation mediators, including leukocytes, complements, antibodies, cytokines, and adhesion molecules contributing to the development of CVD in SLE [16]. Some of the traditional risk factors that are thought to contribute to more premature CVD in our patient include lupus nephritis with progression to end-stage renal disease, lifestyle choices, poorly controlled blood pressure, and use of medication such as steroids for flares.

As cardiovascular complications of SLE are associated with high mortality, medication to improve cardiovascular outcomes is of paramount importance in improving acute and chronic cardiovascular outcomes and lupus-associated mortality. Our patient's home medications include aspirin, statin, anti-hypertensives, seizure medication, and lupus maintenance therapy. Medical management of lupus cardiomyopathy entails two phases: first is the modification of traditional risk factors such as glycemic control, blood pressure control, improving renal function, use of heart failure guideline-directed medical therapy in patients with systolic dysfunctions, use of statins, smoke cessation, and weight control, are important in CVD risk reduction. The second arm of treatment involves the management of SLE-specific risk factors: Several studies demonstrated that low-dose aspirin benefits in protection from arterial thrombosis in all patient groups, with or without anti-phospholipid antibody, although the bleeding risk should be considered [17]. Due to multiple benefits, statins have also been shown to reduce SLE cardiac events by 73% and reduce low-density lipoprotein(LDL) by 29%. So, the cardiovascular benefits of statins in inflammatory diseases are not limited to only lipid-lowering effects but also down-regulation of inflammatory processes, anti-thrombotic, immunomodulation, and stabilization of plaque [18].

HCQ has been recognized as a guideline mainstay for long-term treatment of SLE in all patients due to multiple benefits, except in patients with obvious contraindications. HCQ has shown numerous benefits including suppression of SLE activity, flares, and lowering of long-term glucocorticoid use [19]. It has immunomodulatory effects achieved by interference with autophagosome maturation and obstructs the antigen presentation and lymphocytic activity process. It has also shown numerous favorable benefits to pregnancy and breastfeeding. Several studies demonstrated multiple cardiac benefits of HCQ use in the reduction of atherosclerosis and thrombotic events and the improvement of CVD risk factors in SLE patients [20]. More recent studies have shown that HCQ suppresses proinflammatory cytokine profile, although smoking might inhibit its effects [21]. Consistent use of HCQ in ANA-positive patients delays disease progression and improves disease mortality including renal protection [22]. The benefits to the patient in the current case would be enormous, including suppression of disease activities, renal and other organ protection, or slower progression if complete medication adherence was maintained. Other cardiovascular interventions used in lupus-related cardiovascular disease include biologics such as belimumab, which is widely used for mild to moderate SLE, although long-term cardiovascular benefits have not been studied. Vaccinations, steroids, plasmapheresis, and intravenous immunoglobulins have been scarcely used for acute myocarditis in SLE patients [23].

There have been several reports reviewing HCQ cardiac toxicity. Heart block, ECG changes, heart failure, QT prolongation, QRS widening, cardiomyopathy, and ventricular and atrial arrhythmias have been associated with HCQ use [24]. The majority of reported cases are associated with the use of QT-prolonging antibiotics, especially, for the treatment of coronavirus disease 2019 (COVID-19). In observed solitary HCQ use, some proposed mechanisms of toxicity include pre-existing renal function impairment, for example, lupus nephritis, as it is excreted via the kidney and hepatically. Risk factors for HCQ-induced cardiotoxicity include longer duration, pre-existing cardiac disease, female sex, and renal insufficiency [25]. According to recent systematic studies by Fram et al., cardiac adverse effects rarely occur within the first few years, as the majority of HCQ cardiac toxicity cases occur with high cumulative doses following many years of not monitoring therapy or purposeful acute intoxication. Patients with prior cardiac complications and renal failure may experience more side effects [21]. Furthermore, studies of HCQ cardiac risk association in inflammatory disease reported reduced risk of CVD development in HCQ users and several cardioprotective effects [13]. A new population-based study in 2023, including the entire population of British Columbia, Canada, reported reduced risk of overall cardiovascular events associated with current HCQ use, including a reduction in venous thromboembolism, and cardiovascular preventative benefits [10]. If well supervised while taking recommended doses, with all multidisciplinary care including the cardiologist involved, it is very rare for patients to develop cardiac adverse effects. HCQ safety profile in SLE by Ruiz-Irastorza et al. resulted in a low prevalence of toxicity. A recent meta-analysis for HCQ safety when taken for different pathologies in a daily dose of 200-400 mg gave a high safety profile without cardiac toxicity [11]. Other follow-up measures limiting the occurrence of adverse effects are regular 12-lead ECG and proposed monitoring of cumulative serum level of HCQ, monitoring renal function, and transthoracic echocardiogram, in addition to ophthalmology screening, especially in patients undergoing prolonged treatment. Although the role of HCQ is recognized in SLE, less than half of the patients take HCQ as prescribed even though there is indicated improvement and, in most cases, complete remission of lupus flare and low thrombotic even at a higher normal serum of HCQ [14]. To summarize, HCQ has overwhelming cardiovascular benefits compared to the extremely rare small risk of cardiomyopathy.

Poor disease outcomes in lupus cardiomyopathy, as seen in our patient, can also be partly attributed to several social determinants and health disparities. Health inequities and social determinants of health such as economic, environmental, and psychological factors impact CVD morbidity and mortality [26]. Recent public health disparities have raised awareness of social determinants of cardiovascular health, but research is sparse. Studies show that SLE prognosis has improved over the past four decades, but not equally among racial and ethnic groups [27]. Healthcare disparities exist at all SLE stages including incidence, prevalence, disease activity, damage accumulation, illness manifestation severity, and consequences like cardiomyopathy [27].

The life expectancy of Black SLE patients is significantly lower than White SLE patients due to health disparities. Lack of diversity in research and clinical trials and racial prejudice in patient encounters are noted inequalities. Studies show that Black individuals develop the disease earlier and have more severe symptoms, whereas Hispanic patients have more organ damage than other ethnic groups [27]. Socioeconomic factors are a significant cause of these discrepancies [27]. The African American patient in the current case was from a socioeconomic background marked by low educational attainment, low household income, and impoverished communities. Low socioeconomic level, inequality, and poor cardiovascular health prognosis are highly associated [28]. Lower educational attainment makes disease processes, therapy, and compliance harder to understand [29]. Despite education on compliance, treatment risks, advantages, and outpatient follow-up, the young woman stopped taking her medicine. Significant echocardiographic changes in less than two years imply drug noncompliance may have aggravated her SLE-related cardiac problems. Research shows that approximately 20% of cardiovascular risk is hereditary. Environmental or behavioral factors account for the remaining 80% [30]. Low-income communities often lack sidewalks, parks, playgrounds, and healthy food alternatives.

Access to and affordability of healthcare also affect disease outcomes. Brown et al. showed that primary care clinicians may detect SLE and send patients to specialty care. Lack of easy access to primary or specialist care can lead to delayed diagnosis, inappropriate therapy, inefficient medication, increased complications, organ damage, and higher emergency healthcare use for SLE [31]. Communities with higher disease incidence, prevalence, and severity need more healthcare resources and education. Race has been observed to impact the quality of treatment offered. Fitzgerald and Hurst found that minority ethnic populations got poorer care, fewer specialist referrals, and poorer office communication than White patients [32]. Another study implies clinicians withheld clinical trial treatments and information due to a preconceived perception of minority noncompliance; this worsens outcomes, including multi-organ failure and minority underrepresentation in clinical studies [33]. As shown in the case study, the social determinants of cardiovascular health include many factors that can harm an individual's health, increase the risk and incidence of CVD, and worsen the prognosis for those with these conditions. Understanding socioeconomic determinants of cardiovascular health helps us understand the complexity of societal frameworks and their effects on the circulatory system. If clinicians understand these characteristics, they can reduce preventable disease burdens and health disparities in socially disadvantaged communities. Community and stakeholder mobilization, capacity development, cultural competency, and more financial resources are needed to address individual and community social determinants of health.

SLE also increases the risk of anxiety and depression secondary to extreme pain, chronic exhaustion, sleep issues, and stress. To effectively manage SLE, a comprehensive evaluation is needed to distinguish it from depression [34,35]. Depression due to a medical illness and major depressive disorder have different diagnostic criteria and treatments. Due to overlapping symptoms or perceived somatic complaints, the diagnosis and treatment of depression and anxiety in SLE are typically overlooked or delayed [24]. Research shows that depression and anxiety severity increase SLE disease activity. Mental illness is more likely in SLE patients with Systemic Lupus Erythematosus Disease Activity Index 2000 (SLEDAI-2K) scores above 8.5 [35]. Other studies have demonstrated that SLE, depression, and anxiety increase suicidal ideation and treatment nonadherence, lowering quality of life [36]. Depression and anxiety were observed in our patient, who was non-compliant with her medications or outpatient care. Even though she was not suicidal, our patient's lack of insight into her conditions and poor discernment led to a decline in her mental and cardiovascular health.

Early detection and treatment of depression and anxiety in SLE patients is essential due to their high prevalence and risk. Family income, unemployment, disease activity, and musculoskeletal and neuropsychiatric manifestations affect SLE patients' anxiety and depression. These diverse findings show that biological, social, economic, psychological, and environmental variables may mediate SLE-associated anxiety and depression [35,36]. SLE prognosis and quality of life must be improved by addressing social determinants of mental and cardiovascular health.

## Conclusions

This case report emphasizes the rare cardiac complications that SLE can cause, the available standard treatment, reported benefits and adverse cardiac effects of treatment, the significance of compliance, and the social determinants of cardiovascular health. Lupus-induced cardiomyopathy can cause irreversible injury, necessitating early diagnosis, investigation, and treatment to improve prognosis. This case will add to the literature and supplement the limited data on SLE-associated cardiomyopathy.
